# Modulation of miRNA expression in aged rat hippocampus by buttermilk and krill oil

**DOI:** 10.1038/s41598-018-22148-5

**Published:** 2018-03-05

**Authors:** M. Carmen Crespo, Joao Tomé-Carneiro, Diego Gómez-Coronado, Emma Burgos-Ramos, Alba García-Serrano, Roberto Martín-Hernández, Shishir Baliyan, Javier Fontecha, César Venero, Alberto Dávalos, Francesco Visioli

**Affiliations:** 1 Bioactive Ingredients Food Group, IMDEA Food, Madrid, 28049 Spain; 2 Epigenetics of Lipid Metabolism Group, IMDEA Food, Madrid, 28049 Spain; 3 Área de Bioquímica, Universidad de Castilla-La-Mancha, Toledo, 45071 Spain; 4 Department of Molecular Medicine, University of Padova, Padova, 35121 Italy; 5 Bioactivity and Food Analysis Department, CIAL, CSIC-UAM, Madrid, 28049 Spain; 6 Servicio de Bioquímica Investigación, Hospital Universitario Ramón y Cajal, Madrid, 28034 Spain; 7 CIBER Fisiopatología de la Obesidad y Nutrición (CIBEROBN), Instituto de Salud Carlos III, Madrid, 28029 Spain; 8 Department of Psychobiology, Faculty of Psychology, UNED, Madrid, 28040 Spain

## Abstract

The increasing incidence of age-induced cognitive decline justifies the search for complementary ways of prevention or delay. We studied the effects of concentrates of phospholipids, sphingolipids, and/or 3-n fatty acids on the expression of genes or miRNAs related to synaptic activity and/or neurodegeneration, in the hippocampus of aged Wistar rats following a 3-month supplementation. The combination of two phospholipidic concentrates of krill oil (KOC) and buttermilk (BMFC) origin modulated the hippocampal expression of 119 miRNAs (11 were common to both BMFC and BMFC + KOC groups). miR-191a-5p and miR-29a-3p changed significantly only in the BMFC group, whereas miR-195-3p and miR-148a-5p did so only in the combined-supplemented group. Thirty-eight, 58, and 72 differentially expressed genes (DEG) were found in the groups supplemented with KOC, BMFC and BMFC + KOC, respectively. Interaction analysis unveiled networks of selected miRNAs with their potential target genes. DEG found in the KOC and BMFC groups were mainly involved in neuroactive processes, whereas they were associated with lysosomes and mRNA surveillance pathways in the BMFC + KOC group. We also report a significant reduction in hippocampal ceramide levels with BMFC + KOC. Our results encourage additional in-depth investigations regarding the potential beneficial effects of these compounds.

## Introduction

According to the most recent Global Burden of Disease Study 2015^1^, the burden of neurological disorders has increased substantially over the past 25 years because of expanding population numbers and ageing. Even though the prevalence of communicable neurological disorders decreases, the number of patients who will need care by neurologists will continue to grow in coming decades. Indeed, mostly because of the lack of suitable animal models, neurodegeneration – including cognitive decline - is currently difficult to treat and poses an economical and sociological burden on the national health care systems. Disease-unrelated memory decline increases with age in rodents and humans and appears to be associated with subtle changes in the connectional and functional integrity of key hippocampal circuits^2–4^. Emphasizing the importance of this brain region, several gene transcriptional changes influence a wide range of learning and memory-related processes in the hippocampus of aged rats with memory impairment^5^. Moreover, these processes - including neurogenesis, synaptic plasticity and neuronal connectivity - are affected by nutrition^6^. In this respect, the Mediterranean diet has been suggested as being neuroprotective^7^. Its effects are mediated by changes in the expression of multiple genes and associated regulatory networks; nutrition-gene interactions play important roles in optimal and sub-optimal cognitive function. Epigenetics is also emerging as an important mechanism through which nutrition can directly influence the genome^8^. Numerous epigenetic processes are involved in complex mechanisms of gene regulation, as is the case of synaptic plasticity, learning, and memory. Among other players, these processes include changes in non-protein-coding RNAs (ncRNAs)^8,9^. MicroRNAs (miRNAs), small ncRNAs, which post transcriptionally repress the expression of target genes, have the potential to be used as biomarkers of neurodegenerative disease^10^.

Age-related loss of cerebral n-3 fatty acids (FAs) and polar lipids (PL) together with low intakes of these compounds (which are essential for the activity, functioning, and maintenance of the nervous system) is related to a greater risk of neurodegenerative diseases^11^. The ISSFAL, the World Health Organization (WHO) and the Food and Agricultural Organization (FAO) recommend (for adults) a daily intake of at least 500 mg of eicosapentaenoic acid (EPA) + docosahexaenoic acid (DHA)^12^. In this respect, krill oil is an attractive source of n-3 FAs incorporated into phosphatidylcholine (PC). In addition, buttermilk (BM), a by-product obtained from butter manufacturing with a high content of milk fat globule membrane (MFGM), is rich in polar lipids, particularly phosphatidylserine (PS) and sphingomyelin (SM), with potential positive effects in neurological pathologies^13^ and is currently not employed in the supplement arena. The nutritional recommendations of physiological doses indicated for an adult of phosphatidylserine are 300–500 mg/d.

In this study, we evaluated whether buttermilk and krill oil as sources of essential fatty acids modulate the expression of miRNAs and their target genes in the hippocampus, due to its importance in neurodegenerative processes, of aged rats.

## Results

### Supplementation with polar lipids does not affect body weight

**Figure 1:**
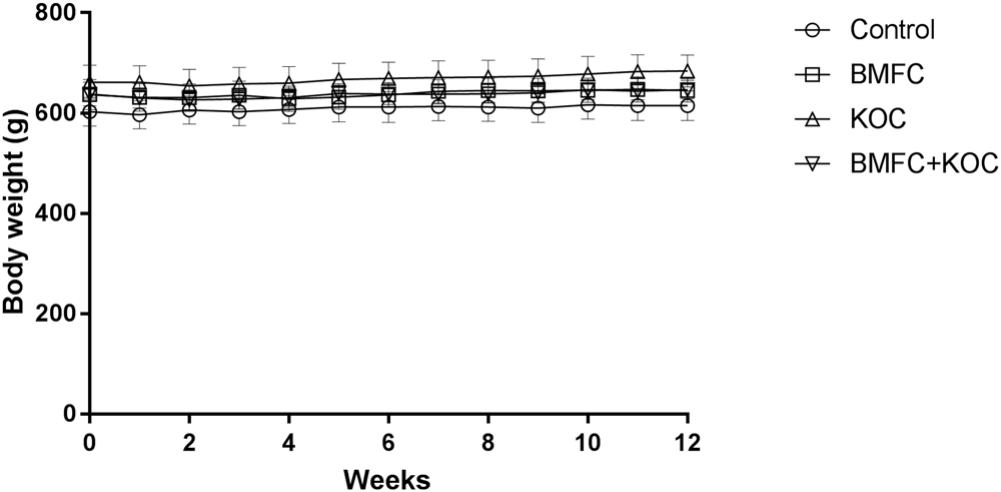
Variation of body mass. Values are expressed as mean ± SEM of the mean. BMFC: buttermilk fat concentrate rich in phospho- and sphingolipids; KOC: krill oil concentrate rich in omega-3 fatty acids (eicosapentaenoic acid and docosahexaenoic acid) and phospholipids; BMFC + KOC: combination of BMFC and KOC.

To determine whether the consumption of different supplements (Table 1) influences body weight, rats were weighted every week during the three months of supplementation. We did not find any significant difference in body weight throughout the study (Fig. 1). The different diets contained similar caloric composition (Table 2) but differ on the types of fatty acids (Table 3).

**Table 1:** Experimental design and administered diets.

*Diets*	Experimental design			
	Control	BMFC	KOC	BMFC + KOC
*standard diet*	EURodent Diet 22%	EURodent Diet 22%	EURodent Diet 22%	EURodent Diet 22%
*Daily jelly*	70 mg refined olive oil	70 mg BMFC	70 mg KOC	70 mg BMFC + 70 mg KOC

BMFC: buttermilk fat concentrate rich in phospho- and sphingolipids; KOC: krill oil concentrate rich in omega-3 fatty acids (eicosapentaenoic acid and docosahexaenoic acid) and phospholipids; BMFC + KOC: combination of BMFC and KOC. EURodent Diet 22% (LabDiet, San Luis, Missouri).

**Table 2:** Nutritional composition of the supplements.

Dose contribution/ animal/Day	Control	BMFC	KOC	BMFC + KOC
Energy (Kcal)	201,07	201,11	201,07	201,75
Lipids (g)	1,82	1,82	1,82	1,89
Carbohydrates (g)	32,10	32,11	32,10	32,11
Fiber (g)	2,05	2,05	2,05	2,05
Proteins (g)	11,01	11,01	11,01	11,01

BMFC, buttermilk fat concentrate rich in phospho- and sphingolipids; KOC, krill oil concentrate rich in omega-3 fatty acids (eicosapentaenoic acid and docosahexaenoic acid) and phospholipids; BMFC + KOC, combination of BMFC and KOC.

**Table 3:** Fatty acids (as methyl esters) included in the nutritional supplements.

MEFA g/100 g supplement	Control	BMFC	KOC	BMFC + KOC
Σ SFA	15,29 ± 0,11	47,77 ± 0,61	27,66 ± 0,27	32,90 ± 0,64
Σ MFA	77,54 ± 0,55	40,20 ± 0,72	23,08 ± 0,15	34,18 ± 1,32
Σ PUFA	6,66 ± 0,16	11,39 ± 0,42	44,73 ± 0,62	33,17 ± 0,83
Σ n-6	6,26 ± 0,10	10,13 ± 0,37	2,09 ± 0,05	5,77 ± 0,50
Σ n-3	0,41 ± 0,06	0.61 ± 0,04	43,06 ± 0,65	24,14 ± 1,07
Σ mFA	0,50 ± 0,10	1,54 ± 0,09	2,22 ± 0,22	2,68 ± 0,07

MEFA, methyl esters of fatty acids; SFA, saturated fatty acids; MFA, monounsaturated fatty acids; PUFA, polyunsaturated fatty acids; n-6, omega−6 fatty acids; n-3, omega−3 fatty acids; mFa, other minor fatty acids; BMFC, buttermilk fat concentrate rich in phospho- and sphingolipids; KOC, krill oil concentrate rich in omega-3 fatty acids (eicosapentaenoic acid and docosahexaenoic acid) and phospholipids; BMFC + KOC, combination of BMFC and KOC.

### Blood tests

**Figure 2:**
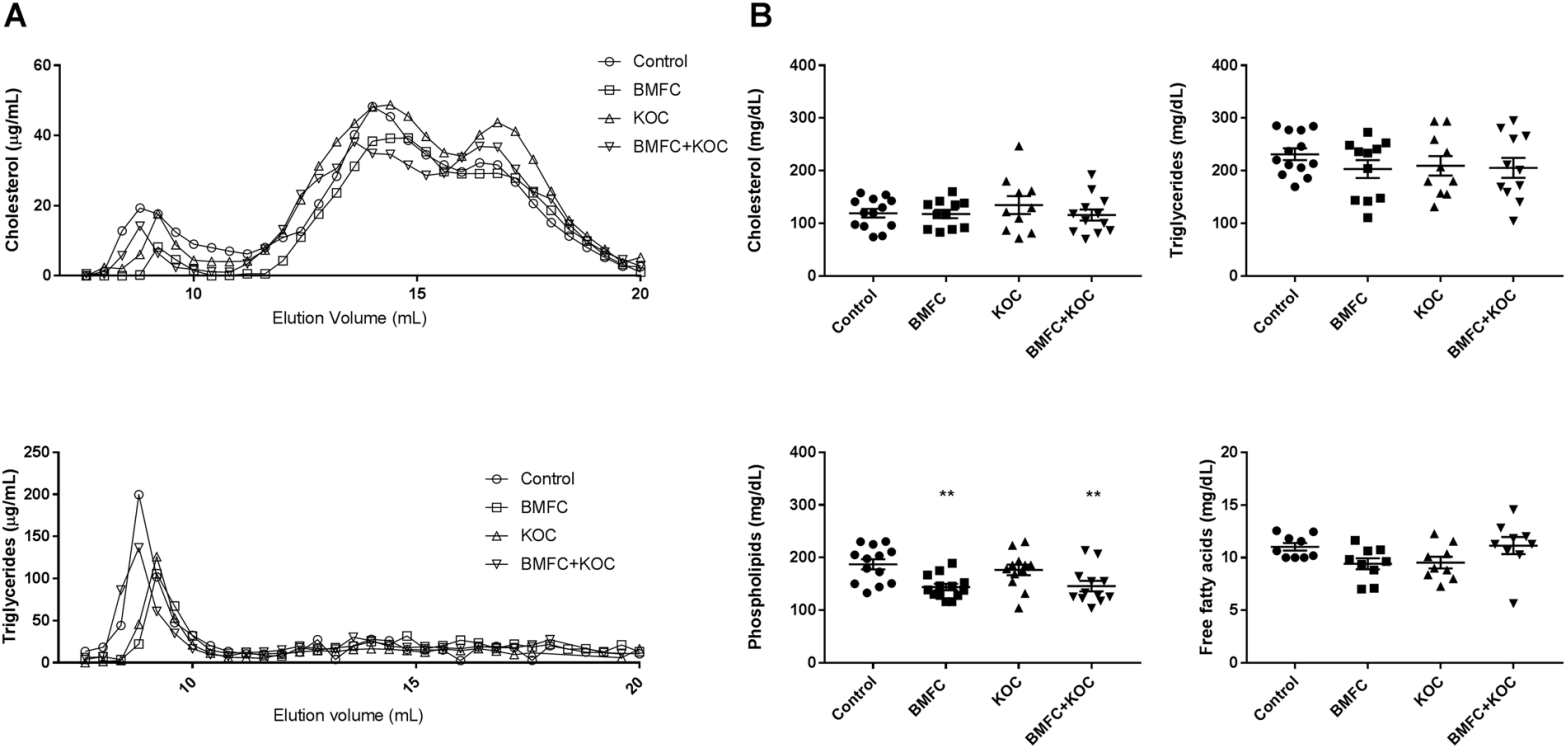
Lipid levels and lipoprotein profile of rat plasma. (**A**) FPLC profile of plasma cholesterol and triglycerides. (**B**) Cholesterol, triglycerides, non-esterified fatty acids, and phospholipid levels in plasma. Values are means ± SEM. Statistically significant difference from control at *p < 0.05–0.005; **p < 0.005–0.0005. BMFC: buttermilk fat concentrate rich in phospho- and sphingolipids; KOC: krill oil concentrate rich in omega-3 fatty acids (eicosapentaenoic acid and docosahexaenoic acid) and phospholipids; BMFC + KOC: combination of BMFC and KOC.

We studied selected biological markers to see if supplements had effects on the lipid profile, namely on cholesterol, triglyceride, non-esterified fatty acid, and phospholipids concentrations. There were no significant differences between the groups in the lipoprotein profile as analyzed by FPLC (Fig. 2A); significant differences were found in phospholipid levels, which were decreased in the BMFC and BMFC + KOC groups as compared with controls (Fig. 2B). These decreases could be due to a compensatory physiological mechanism in response to the extra supply of phospholipids. We did not find significant changes between groups in the other parameters analyzed.

### Ceramide levels are reduced by BMFC + krill oil supplementation

**Figure 3:**
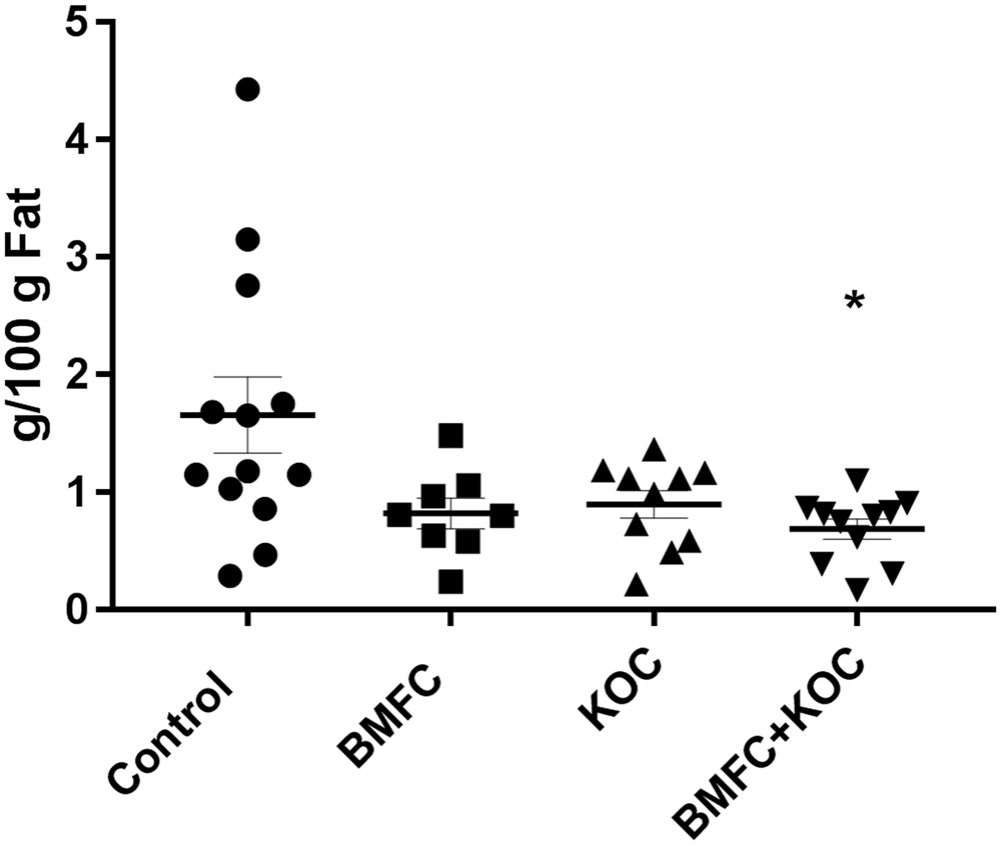
Ceramide concentrations in the hippocampus. Values are the mean ± SEM, n ≥ 10 per group. Different letters denote differences at *p* < 0.05. BMFC: buttermilk fat concentrate rich in phospho- and sphingolipids; KOC: krill oil concentrate rich in omega-3 fatty acids (eicosapentaenoic acid and docosahexaenoic acid) and phospholipids; BMFC + KOC: combination of BMFC and KOC.

Possible effects of dietetic supplementations on the composition of key lipids were assessed in the hippocampus. No major differences in lipids classes were observed (results not shown), except for ceramides, whose level was significantly reduced in the BMFC + KOC group (Fig. 3).

### Supplementation with phospholipid concentrates modulates the expression of selected miRNAs

**Figure 4:**
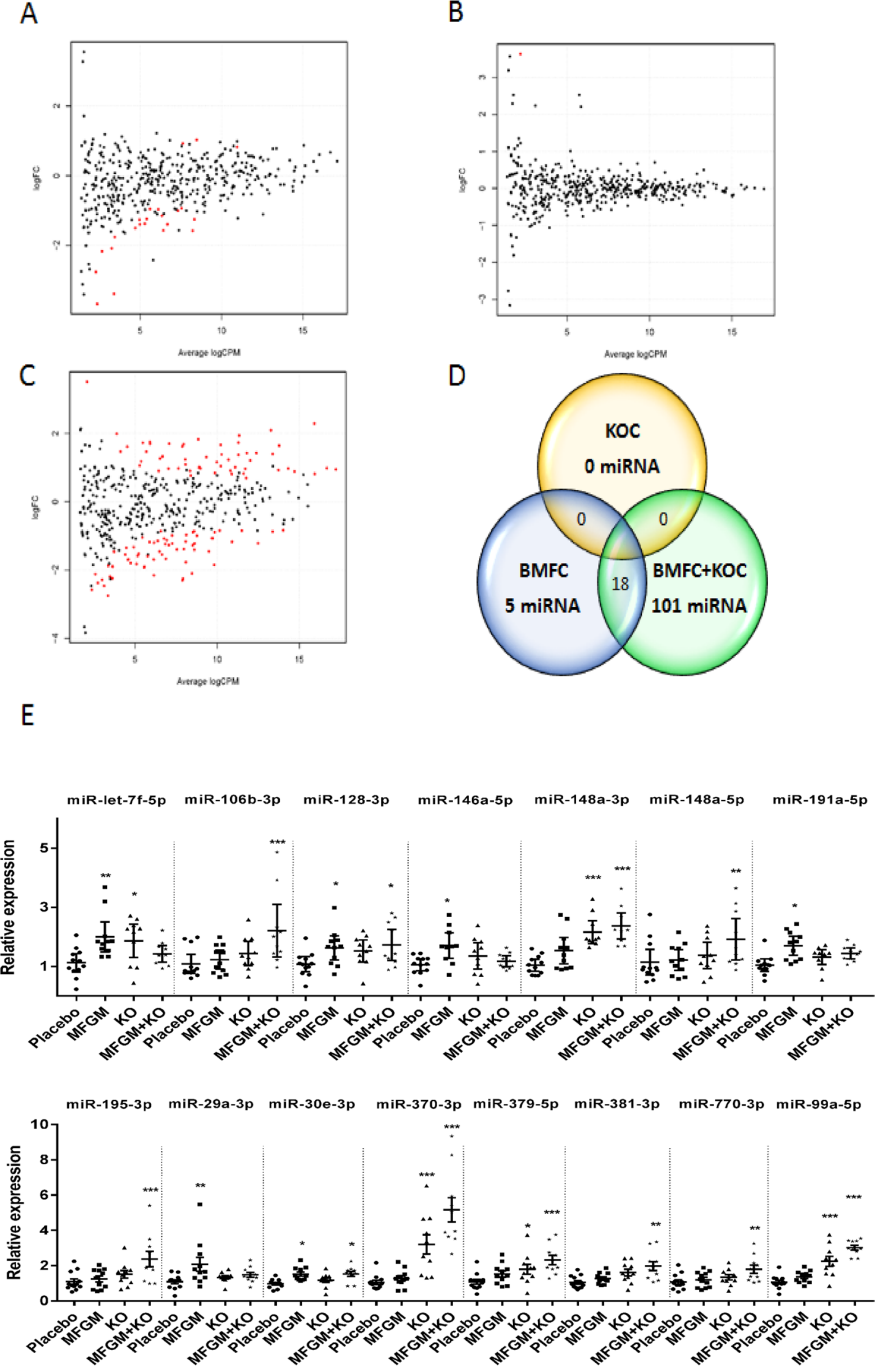
sRNA_Seq_ results. Smear plots for control vs KOC (**A**), BMFC (**C**) and KOC + BMFC (**B**) groups. (**D)** Venn diagram of differentially expressed miRNAs. (**E)** Validation of selected miRNAs. Values are means ± SEM. Statistically significant difference vs control at *p < 0.05–0.005; **p < 0.005–0.0005; ***p < 0.0005. BMFC: buttermilk fat concentrate rich in phospho- and sphingolipids; KOC: krill oil concentrate rich in omega-3 fatty acids (eicosapentaenoic acid and docosahexaenoic acid) and phospholipids; BMFC + KOC: combination of BMFC and KOC.

As epigenetic regulation of gene expression is highly flexible in the brain^14^ and miRNAs – considered epigenetic markers – are involved in brain development and cognition^10^, we assessed the expression of miRNAs in the hippocampus. Whole transcriptome of small RNAs was performed by RNA-sequencing. We show that numerous miRNAs were induced or repressed in response to phospholipid concentrates supplementations (Supplementary Table S1). Compared to the control group, BMFC differentially modulated the expression of 23 miRNAs (Fig. 4A), three up-regulated and 20 down-regulated, whereas krill oil did not exert any significant effects (Fig. 4B). Interestingly, the mixture of both lipids dramatically modulated the expression of these and other novel miRNAs. Namely, 119 miRNAs were modulated: 55 of them overexpressed and 64 repressed (Fig. 4C); 18 miRNAs are shared between BMFC and BMFC + KOC (Fig. 4D).

In order to confirm our results, we selected some miRNAs and validated them using RT-qPCR. Fifteen miRNAs were selected based on criteria such as the level of change in expression and their involvement in processes of the nervous system (Supplementary Table S2). Validation of the selected miRNAs of both treatments indicated that BMFC and BMFC + KOC modulate the expression of miRNAs in the hippocampus (Fig. 4E).

Validation confirmed that both BMFC and BMFC + KOC supplementation modulated the expression of 11 miRNAs in the hippocampus: miR-99a-5p, -128-3p, -148a- 3p, -379-5p, -381-3p, -146a-5p, -30e-3p, -370-3p, -106b-3p, -770-3p and let-7f-5p (Fig. 4E). On the other hand, miR-191a-5p and -29a-3p showed significant changes only in rats supplemented with BMFC; while miR-195-3p and -148a-5p were the only miRNAs significantly affected in the combined-supplement group. Moreover, miR-148a-3p, -370-3p, -379-5p, -99a-5p and let-7f-5p showed a significant expression increase in KOC-consuming rats, although no significant changes had been found by mass sequencing. In addition, miR-128-3p and -30e-3p were up-regulated both in the BMFC group and in the combined-supplement fed group.

With regards to the significant miRNAs validated by RT-qPCR, functional analysis of their predicted target genes showed an association with nervous system related processes, such as axon guidance, neurotrophin signaling, neuroactive ligand-receptor interaction, neuron differentiation and migration or neurological development, among others (Supplementary Table S3).

### Supplementation with phospholipid concentrates modulates hippocampus gene expression

**Figure 5:**
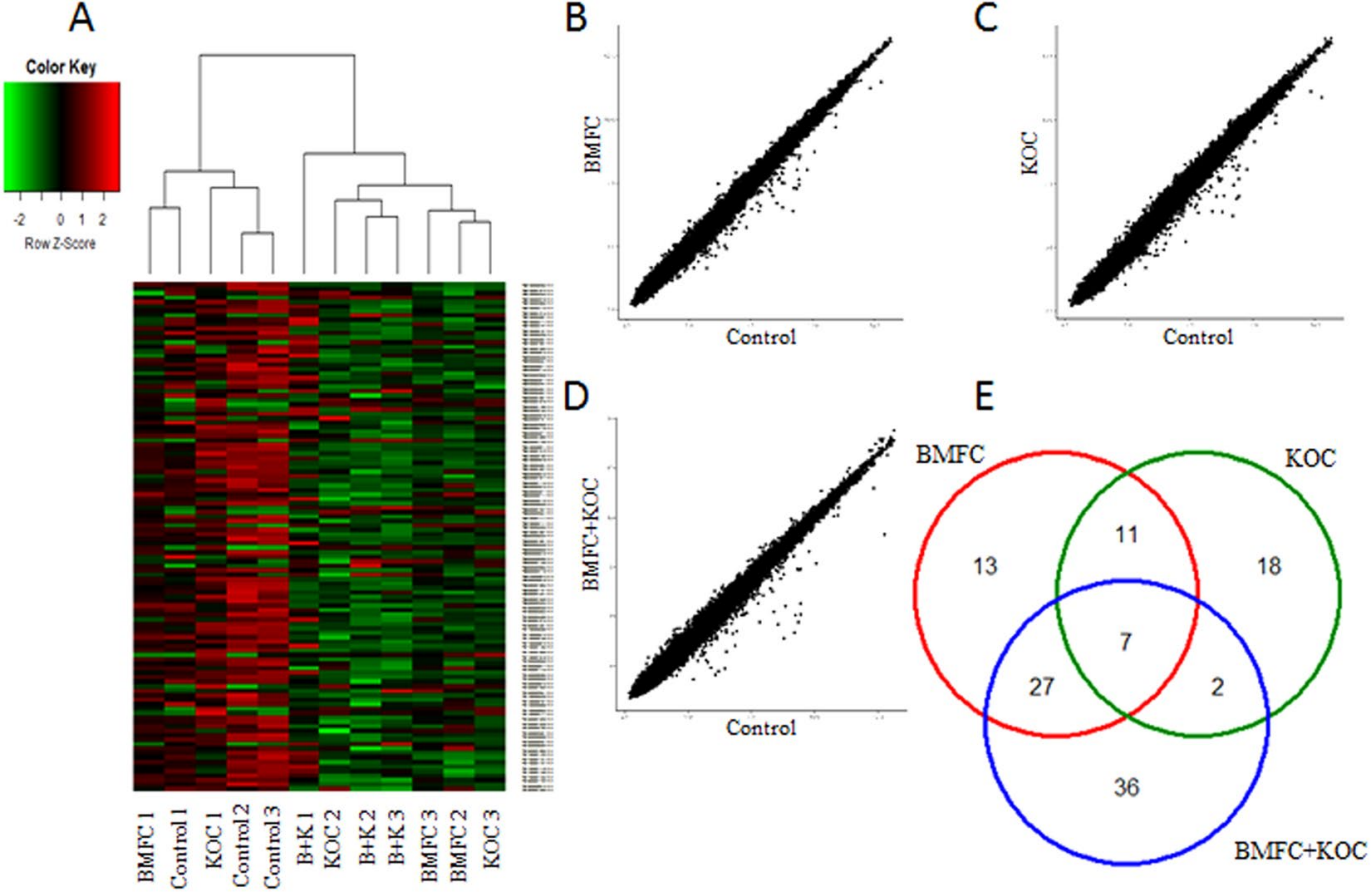
Gene expression analysis. (**A**) Heatmap of microarray data. Scattered plots for KOC (**B**), BMFC (**C**) and KOC + BMFC (**D**) groups. (**E**) Venn diagram of differently expressed genes. BMFC: buttermilk fat concentrate rich in phospho- and sphingolipids; KOC: krill oil concentrate rich in omega-3 fatty acids (eicosapentaenoic acid and docosahexaenoic acid) and phospholipids; BMFC + KOC: combination of BMFC and KOC.

To appraise the diet-modulated hippocampal transcriptome, we performed gene expression microarrays. The complete results are shown in Supplementary Table S4. The Heat Map representation of each of the three supplemented groups is shown in Fig. 5A and dispersion representation in Fig. 5B–D.

Thirty-eight genes were found to be differentially expressed in the KOC supplemented group, 11 up-regulated and 27 down-regulated. In the group supplemented with BMFC, we found 58 differentially expressed genes (DEG), two of which with higher expression and 56 with lower expression than in the control group. Finally, the group that consumed the BMFC + KOC diet had the highest number (72) of DEG, of which 11 were up-regulated and 61 down-regulated. Some DEG were shared by two or three groups (Fig. 5E).

Functional analysis of the differently expressed genes found in the microarray was based on different databases and displayed several neural system-related processes, among others (Supplementary Table S5). In all supplemented groups, we found processes related to lipid metabolism, like fatty acids synthesis and arachidonic acid metabolism, and neurotransmitter biosynthesis, such as serotonin, adrenaline and noradrenaline, transport and brain development.

### BMFC-modulated genes in the hippocampus are influenced by miRNA-gene interaction

**Figure 6:**
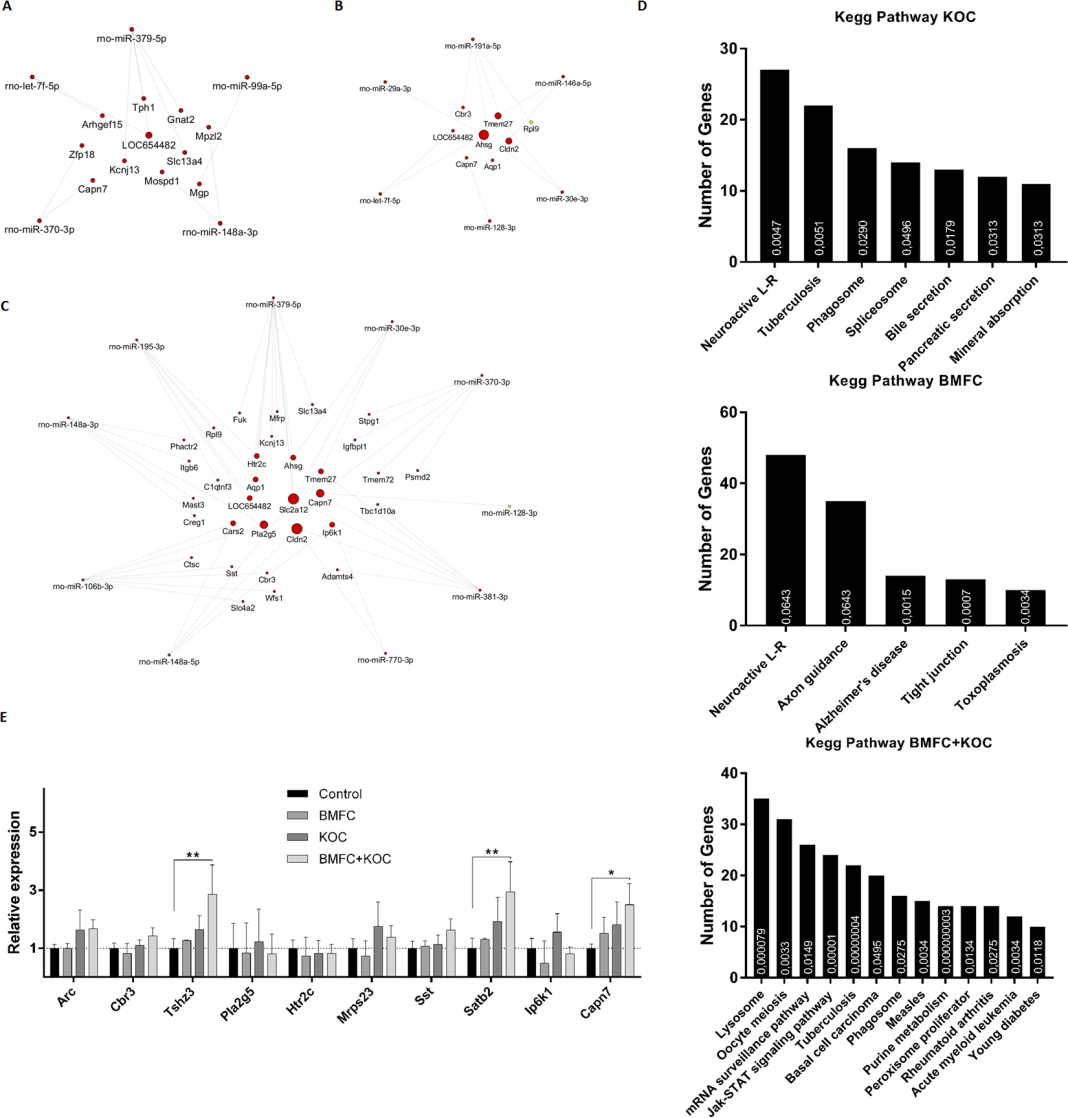
Genetic interaction analysis between miRNAs and their possible mRNAs targets for KOC (**A**), BMFC (**B**) and BMFC + KOC (**C**) groups. (**D**) Functional enrichment analysis of differentially expressed genes. (**E**) Validation of potential target genes of previously validated miRNAs. Arc: activity-regulated cytoskeleton-associated protein; Cbr3: carbonyl reductase 3; Tshz3: teashirt zinc finger homeobox 3; Pla2g5: phospholipase A2 group V; Htr2c: 5-hydroxytryptamine receptor 2 C; Mrps23: mitochondrial ribosomal protein S23; Sst: somatostatin; Satb2: SATB homeobox 2; Ip6k1: inositol hexakisphosphate kinase 1; Capn7: calpain 7. BMFC: buttermilk fat concentrate rich in phospho- and sphingolipids; KOC: krill oil concentrate rich in omega-3 fatty acids (eicosapentaenoic acid and docosahexaenoic acid) and phospholipids; BMFC + KOC: combination of BMFC and KOC.

Due to the post-transcriptional regulation by miRNAs, we performed an interaction analysis between DEG identified in the array and the miRNAs selected for validation (Supplementary Table S6). This analysis related the modulated miRNAs and their targets, considering their regulatory function, i.e. up-regulated miRNAs with down-regulated targets or vice versa. In the KOC group, five miRNAs formed a network of interaction with 11 DEG (Fig. 6A). In the BMFC group, six miRNAs showed an interaction network with eight DEG (Fig. 6B). Finally, in the BMFC + KOC group, 32 genes were found to be potential targets of 10 miRNAs (Fig. 6C). According to gene ontology analysis (Fig. 6D), DEG found in the group consuming KOC are primarily involved in receptor-ligand neuroactive processes, although an involvement in transport processes such as secretion and absorption was also identified. Neuroactive receptor-ligand processes were also the main route found in the BMFC group, together with axonal orientation and Alzheimer’s disease-related processes. As for the BMFC + KOC group, DEG were associated with lysosomes - cells’ digestive system, and mRNA surveillance pathways, a quality control mechanism to detect and degrade abnormal mRNAs. DEG found in this group were also involved in transport processes such as secretion and absorption.

Ten DEG were then selected for validation by RT-qPCR, based on their association with cognitive function/neurodegenerative disease, and on the condition that they were predicted targets of miRNAs previously associated to some type of cognitive function (Supplementary Table S6). Three up-regulated genes - *Tshz3*, *Satb2* and *Capn7*- were validated in the BMFC + KOC group with confirmation of statistically significant changes compared to the control group (Fig. 6E).

## Discussion

We supplemented aged rats with a MFGM-rich concentrate and/or a krill oil concentrate for three months and report the transcriptomic and miRNA expression in their hippocampus, in addition to changes in total ceramide concentrations.

Previous works have shown that MFGM supplementation of human infants (6–11 months of age) could reduce diarrhea episodes^15^ or acute otitis media infections^16^, which might be related to oral microbiome changes^17^. MFGM have also shown to reduce differences in cognitive development^18^ and serum lipid status^19^ between formula- and breast-fed infants (from < 2 until 6 months of age) when supplemented in fed-formula. Interestingly, young piglets supplemented with MFGM and other prebiotics seemed to be more advanced neurodevelopmentally^20^. In adults (18–65 y of age), buttermilk supplementation reduced cholesterol concentrations^21^ and blood pressure in normotensive individuals^22^. Indeed, supplementation of overweight adults with milk fat enclosed in MFGM prevented the increase of plasma lipids compared to that of milk fat without MFGM^23^. Regarding other physiological contexts, consumption of MFGM (1 g/day during 10 weeks) plus exercise have been shown to improve physical performance^24^ or skeletal muscle strength (1 g/day during 4 weeks)^25^ of healthy adults. In the elderly, dietary supplementation with MFGM (1 g/day during 3 months) combined with exercise improved the frailty status as compared to the MFGM alone^26^. Moreover, also in elderly (60–73 y), supplementation with MFGM (1 g/day for 10 weeks) was shown to increase physical performance and muscle function when coupled with a light exercise program^27^. However, very few studies focused on brain function^18,20^ and none on the aged brain.

We did not observe any appreciable adverse effect due to dietary supplementation. Although MFGM consumption by healthy adults has been shown to be safe at a doses of 6.5 g for 4 weeks^28^, a higher rate of eczema was observed in infants administered MFGM-enriched infant formula supplementation (up to 4 months of age) as compared with standard infant formula^29^.

To the best of our knowledge, our study is the first to evaluate miRNA expression in response to BFMC supplementation. Dietary modulation of miRNAs, i.e. through food bioactive compounds^30^ or dietary lipids^31,32^, is feasible and might be an alternative to the pharmacological modulation of miRNAs. Through gene interaction analyses, we report that miRNAs might contribute to the regulation of the modulated hippocampal genes. Gene Ontology analysis suggests that modulated genes participate in pathways related to neuroactive receptor-ligand processes, axonal orientation, Alzheimer’s disease-related processes, or lysosomes pathways, most of which are related to brain function. Some such modulated genes have been previously associated with synaptic activity. Three genes – *Capn7*, *Tshz3* and *Satb2* – coding for proteins involved in neurological processes were confirmed to have increased expression in the BMFC + KOC supplemented group compared to control. For example, although the physiological importance of Capn7 is not clear, calpains are ubiquitous calcium sensitive proteases involved in essential neuronal functions including maintaining synaptic plasticity, protein turnover and cell signaling^33^. In addition, Tshz3 deletion affects the cortical expression of a number of genes related to autism spectrum disorder (ASD) and induces ASD-relevant deficits that are associated with functional changes at synapses formed by deep-layer cerebral cortical projection neurons (CPN)^34^. Finally, Satb2 is necessary for long-term memory formation and hippocampal late-long-term potentiation and determines the expression of protein-coding genes and miRNAs linked to learning and memory in the hippocampus^35^. In this context, the increased expression seen in these genes, in the BMFC + KOC supplemented group, could be beneficial for well-balanced neurocognitive processes.

Even though whether the modulated genes are only a direct consequence of the modulated miRNAs it is still unclear, we show that BFMC + KOC supplementation influences the expression of several mRNAs and miRNAs in the hippocampus. In this sense, a general association with nervous system related processes, such as axon guidance, neurotrophin signaling, neuroactive ligand-receptor interaction, neuron differentiation and migration or neurological development, was found in the functional analysis of the RT-qPCR-validated miRNAs. Together with the above-mentioned genes, several miRNAs described herein have been previously related to neurodegenerative diseases. For example, it has been proposed that a loss of specific miRNAs, such as the cluster miR-29a/b-1, can contribute to increased BACE1 and Aβ levels in sporadic Alzheimer’s disease^36^. If this is the case, then the increase in miR-29a expression seen in the BFMC-supplemented group could be advantageous against AD, nonetheless additional investigation is required. On the other hand, Lukiw *et al*. suggested that miRNA-146a-mediated modulation of complement factor H (an important repressor of the inflammatory response of the brain) gene expression may in part regulate an inflammatory response in AD brain and in stressed HN cell models of AD^37^. In this context, the rise in miR-146a expression seen in the BFMC group could be related to changes in inflammatory processes; again, further ad-hoc research is needed. Also, Kim and colleagues identified miR-106b as a regulator of Aβ metabolism, increasing Aβ production and preventing its clearance. In this case, the rise in miR-106 observed for the BMFC + KOC supplemented group does not appear to be desirable, but literature is scant and additional studies are indispensable. We acknowledge that additional investigation is necessary. Moreover, despite the modulation of the aforementioned miRNAs, rises in expression levels could not be surely correlated with a decrease in their respective predicted target genes validated here and causality could not be established. In this context, gene expression analyses reveal changes in mRNA levels even though miRNAs can bind to mRNAs without necessarily inducing degradation but still being able to prevent protein synthesis, which could have an important impact on cell function. In fact, we cannot discard other interactions, i.e. the case of a miRNA that impacts on the protein expression levels without any apparent changes on its mRNA target, due to the solely evaluation of the transcriptome (and not protein levels). Overall, we believe that our results warrant further investigation to determine the impact of these supplements on cognitive symptoms associated with aging, that is to say more in-depth biological/molecular analysis and behavioral tests are justified. Indeed, a major limitation of this work is that we did not performed any behavioural test or biochemical analysis on brain tissue that validate the functional effects of our results showed at genomic levels.

Consumption of omega-3 fatty acids from marine sources^38^ or their derivatives^39^ decreases plasma triglyceride levels. Namely, krill oil consumption decreases triglyceride concentrations^40^ both in healthy^41^ and hypertrygliceridemic subjects^42^. The bioavailability of n-3 FAs from krill has been shown to be similar to that of other long-chain n-3 polyunsaturated fatty acids^43^; however, their specific effects in the elderly have not been studied thus far. A recent study suggested that dietary krill oil supplementation enhanced neurocognitive function in aging mice, by changing the expression of Celsr3 and Ppp1r1b^44^, which are implicated in memory and learning process. In this study, we did not observe any change in triglyceride concentrations in supplemented aged rats. Whether krill oil or other sources of omega-3 fatty acids also influence lipid levels in the elderly remains unresolved. Indeed, omega-3 fatty acid-rich oils (beginning at 12 months of age) apparently shorten the life span of long-lived F1 mice and krill oil modestly increases bilirubin, triglyceride, and glucose levels^45^. Whether the lowering effects of omega-3 fatty acid-rich oils on triglyceride levels are reduced by aging is unknown.

We also report that dietary supplementation with different phospholipids did not remarkably influence the lipid profile of the hippocampus, with the exception for a significant reduction in ceramide levels in rats supplemented with a MFGM-rich concentrate in combination with a krill oil concentrate. Whether changes in ceramide levels can contribute to delaying mild-cognitive decline due to aging is not clear but further investigation is entitled. Ceramides play an important role in the neurons and its composition changes through the different stages of development^46^. Indeed, ceramide accumulations in the brain results in a deregulation of energy balance and lead to different disorders as obesity, disturbance of glucose homeostasis^47^, neurodegenerative diseases^48^ and AD pathogenesis^49,50^. Our data shows changes in the transcriptomic and miRNA expression profiling in response to dietary supplementation with a MFGM-rich concentrate in the hippocampus. Whether there is a direct causality between these changes and the reduction in ceramide levels is unknown, but, to the best of our knowledge, cannot be discarded. Ceramide deregulation has been shown to cause distinct global alterations of gene expression in hepatocyte cell lines^51^ or changes in miRNA expression in multiple myeloma cells *in vitro*^52^. However, the *in vivo* effects of these changes on the whole transcriptome or miRNome are understudied, especially in the hippocampus.

In summary, the experimental evidence presented here and elsewhere suggests that a buttermilk fat concentrate, rich in MFGM, alone or in combination with a krill oil concentrate, may influence cognitive development^18^ or brain function^20^ by targeting both genetic and epigenetic mechanisms through miRNA modulation. Whether these epigenomic and nutrigenomic changes can influence functional readouts is still unknown. As mentioned in the Introduction, the increasing incidence of age-induced cognitive declines calls for immediate action and the use of dietary supplements, e.g. BFMC + KOC and others warrants intense investigation.

## Material and Methods

### Animals, diets and experimental design

We followed the Guide to the Care and Use of Laboratory Animals, published by the US National Research Council (Eight Edition, 2010). The animal experimentation committee of the National University of Distance Education (UNED) approved these experiments. Nine-month old Wistar rats (n = 46) were purchased from Charles River Laboratories (Barcelona, Spain) and were housed for nine months. At 18 months of age (when rats begin to present a variety of cognitive symptoms associated with aging), the animals were randomly assigned to four experimental groups of diet supplementation with phospho- and sphingolipids concentrates from buttermilk (BM) and krill oil (KO), or a combination of both (Table 1). Concentrates were produced at the Institute of Food Science Research (CIAL, Madrid, Spain) and were given in the form of frozen strawberry jellies. Nutritional and methyl ester fatty acids composition of the concentrates are described in Tables 2 and 3, respectively. The nutritional intervention lasted for three months. Briefly, rats were weighed every week during the supplementation period, after which they were sacrificed by decapitation. Blood samples were centrifuged for plasma collection at 1500 xg for 15 minutes, RT. Tissues and organs were quickly extracted and frozen in liquid nitrogen. All samples were stored at −80 °C.

### Determination of plasma lipids and lipoproteins

Plasma concentrations of cholesterol and triglycerides were analyzed enzymatically using commercial kits (Centronic, Germany & Bradford Diagnostics, Kemia Científica S.A., Spain). To determine the plasma lipoprotein profile, a pool of plasmas was subjected to fast protein liquid chromatography (FPLC) gel filtration through a Superose 6 HR 10/30 column (Pharmacia). Samples (220 μl) were eluted with 150 mM NaCl, 10 mM Tris–HCl, 2 mM Na2-EDTA and 0.02% NaN3, pH 7.4, at a flow rate of 0.3 ml/min, and 0.4-ml fractions were collected.

Cholesterol, triglyceride, non-esterified fatty acid (FAs) and phospholipid levels were determined in plasma samples using appropriate commercial assay kits, according to the instruction’s manual (Spinreact, Sant Esteve de Bas, Spain & Wako Chemicals GmbH, Neuss, Germany).

### Ceramide and lipid analysis

Tissue lipids were extracted using the Folch method described by Löfgren *et al*.^53^ with slight modifications. Briefly, tissues samples were dissolved in methanol in 50 mL glass tubes. The mix was sonicated in an ultrasonic processor (Dr. Hielscher, Teltow, Germany) during 15 second, two cycles. Then, dichloromethane (1:2 methanol/dichloromethane) was added and mixed during 20 min. Acetic acid 20 mM (1:3 acetic acid/dichloromethane) was added and samples were again mixed for 20 min. Samples were centrifuged at 2100 rpm, 5 min, 4 °C. Bottom organic phases were transferred to a new glass tube and the methanolic phase was washed with dichloromethane (1:1 dichloromethane/methanol) and mixed for 10 min before centrifugation with the same conditions as described above. The organic phases were collected and mixed and filtered through 0.45 µm, evaporated with nitrogen and weighted. Lipids extracts were maintained at −35 °C. The separation of ceramides of lipid extracts from tissues samples were performed with a HPLC Agilent Technologies, model 1200 (Agilent Technologies, Palo Alto, CA, USA) coupled to an evaporative light scattering detector (ELSD) (SEDERE. SEDEX 85 model, Alfortville Cedex, France) using pre-filtered compressed air as the nebulizing gas at pressure of 350 KPa, temperature of 90 °C and the gain was set at 6. Two columns Zorvax Rx-SIL column (Agilent Technologies, Palo Alto, CA, USA) of 250 mm × 4.5 mm and 5 μm particle size, were coupled in series with a precolumn with the same refill. They were equilibrated at 40 °C. The injection volume was 50 μL at concentration of 5 mg/mL in CH_2_Cl_2_ using chromatographic solvent gradient as we have previously described^54^.

### RNA isolation

Total small and large RNA extraction from hippocampus samples were performed according to the manufacturer’s instructions (NucleoSpin^®^ miRNA kit, Macherey Nagel, Düren, Germany). RNA quantity was determined by NanoDrop® ND-2000 Spectrophotometer (Thermo Fisher Scientific Inc., Spain).

### Small RNA library construction and sequencing

Purity and integrity of RNA extracted from hippocampal samples (*n* = 5 per group), were assessed with an Agilent 2100 Bioanalyzer (Agilent Technologies, Santa Clara, CA). A NEBNext® Small RNA Library Prep Set for Illumina (New England BioLabs, Ipswich, MA) was used to prepare the libraries according to the manufacturer’s protocol. The cDNA library was sequenced with NextSeq. 500 from Illumina. To facilitate the analysis a filter of sequences with a maximum length of 50NT was applied. Samples passed the quality controls performed through the FASTQC tool. Bowtie2 was used for sequence alignment with reference genomes, first against rat miRNA sequences downloaded from miRbase, and then against the rat genome Rnor_5.0 (Ensembl release 79). The integrated Genome Viewer (IGV) software was used for alignment visualization. Finally, gene counting was carried out using HTSeq-count to allow differential expression analysis, mainly, of the miRNAs obtained from each experimental group versus the control group.

### Arrays

Expression arrays were performed in hippocampal tissue of three samples per study group, using the 4 × 44 K complete rat genome platform from Affymetrix (Clariom S Assays). Each microarray screens approximately 22,900 unique rat genes and transcripts. Briefly, double-stranded cDNA was synthesized from total RNA using One-cycle cDNA Synthesis Kit (Affymetrix, Santa Clara, CA). cRNA was fragmented and hybridized to the Affymetrix matrix following the manufacturer’s instructions. Finally, microarrays were washed and cartridges were scanned in a GeneChip® 3000 scanner for fluorescence signal acquisition.

The generated output files in CEL format were used for downstream analysis. Data was normalized with the RMA method. Probes with lower variance were filtered out, resulting in approximately 12000 remaining probes. Differential gene expression was assessed with the Bioconductor Limma package. Genes with a FDR lower than 0.1 were considered as statistically significant.

### Interactions analysis

For interaction analysis, we screened miRNAs selected for validation. Prediction of miRNA targets was obtained using the Diana microT-CDS algorithm. Only the corresponding targets that showed statistically significant differential gene expression in microarrays were considered as valid interactions. Target point sizes are directly correlated with the number of interactions within the set of miRNAs.

### Functional analysis and miRNA target prediction

Genecodis3 algorithm was used for functional analysis of the obtained gene lists, focusing on biological processes and pathways, using the Gene Ontology and KEGG pathway databases respectively. Predicted miRNA gene targets were obtained using the Diana microT-CDS algorithm. For each miRNA, the prediction score was set to a convenient value in order to obtain in the output a gene target list ranging from 200 to 1500 items.

### Validation of miRNA and gene expression by RT-qPCR

miScript®II reverse transcription kit (Qiagen, Germantown, MD) was used to converted total RNA into first strand cDNA for miRNA validations whereas iScript™ Advanced cDNA Synthesis Kit (BIO-RAD, Hercules, CA) was used for gene validations, both according to the manufacturer’s guidelines.

Selected miRNAs were obtained from the miRBase database, and the genes for the validation were designed using Primer3 online program and amplify4 (Supplementary Tables S7 and S8). Designed primers were constructed by ISOGEN (Life Science, Belgium). Real time PCR was performed in a 384-plate format using a 7900HT system (Life Technologies, Alcobendas, Spain). Cycling conditions for both gene and miRNA amplification included a first step of activation at 95 °C 15 minutes. For gene amplification, 40 cycles at 94 °C for 15 seconds for the denaturation, annealing at 58 °C for 30 seconds were applied subsequently. As for miRNA amplification, 40 cycles of denaturation at 94 °C for 15 seconds, annealing at 58 °C for 30 seconds and an extension at 70 °C for 15 seconds were applied. Finally, a dissociation curve step at 95 °C for 15 seconds, followed by 15 seconds at 60 °C and 95 °C for 15 seconds was carried out for both amplification types. miScript SYBR Green qPCR Master Mix (Qiagen,Madrid, Spain) was used for both validations. GAPDH and RNU6 were used as reference genes for gene and miRNA data normalization, respectively. The ΔΔCt method was used for gene expression analysis and fold-change values were reported as 2^−(ΔΔCt)^.

### Statistical analysis

Significance of the functional enrichment results was assessed using the hypergeometric test and items with corrected *p* < 0.05 were considered as statistically significant. Differences between groups were assessed by one-way ANOVA followed by Bonferroni test for post hoc comparisons. *p* < 0.05 was considered as statistically significant. Results are presented as means ± SEM. Statistical analyses were performed with GraphPad Prism 7.02 software (GraphPad Software, Inc., La Jolla, CA, USA).

## Electronic supplementary material

Supplementary Information

Table S3

Table S5
