# Supplementary material for: Modulation of miRNA expression in aged rat hippocampus by buttermilk and krill oil
Source: Sci Rep. 2018 Mar 5;8:3993. doi: 10.1038/s41598-018-22148-5 (PMC5838111; doi:10.1038/s41598-018-22148-5)
Supplement: Supplementary file 1 — Supplementary Information [file 41598_2018_22148_MOESM1_ESM.pdf]

Modulation of miRNA expression in aged rat hippocampus by buttermilk and krill oil

M. Carmen Crespo[1§], Joao Tomé-Carneiro[1§], Diego Gómez-Coronado[6,7], Emma Burgos-Ramos[3], Alba García-Serrano[5], Roberto Martín-Hernández[1], Shishir Baliyan[8], Javier Fontecha[5], Cesar Venero[8], Alberto Dávalos[2*], Francesco Visioli[1,4*]

[1]Bioactive Ingredients Food Group, IMDEA Food, Madrid-28049, Spain.

[2]Epigenetics of Lipid Metabolism Group, IMDEA Food, Madrid-28049, Spain.

[3]Área de Bioquímica, Universidad de Castilla-La-Mancha, Toledo-45071, Spain.

[4]Department of Molecular Medicine, University of Padova, Padova-35121, Italy.

[5]Bioactivity and Food Analysis Department, CIAL, CSIC-UAM, Madrid-28049, Spain.

[6]Servicio de Bioquímica Investigación, Hospital Universitario Ramón y Cajal, Madrid-28034, Spain.

[7]CIBER Fisiopatología de la Obesidad y Nutrición (CIBEROBN), Instituto de Salud Carlos III, Madrid-28029, Spain.

[8]Department of Psychobiology, Faculty of Psychology, UNED, Madrid-28040, Spain.

[§]These authors contribute equally to this work.
Corresponding author:
Alberto Dávalos
Madrid Institute for Advanced Studies (IMDEA)-Food, Madrid, Spain
e-mail: alberto.davalos@imdea.org (AD)

Supporting Information

Additional supporting information may be found in the online version of this article.

Table S1. miRNA sequencing results.

Table S2. Selected miRNAs for validation by RT-qPCR.

Table S3. Functional analysis of the significant miRNA of NGS validated by PCR. (Excel File)

Table S4. Gene expression microarray results.

Table S5. Functional analysis of the significant genes of the array. (Excel File)

Table S6. Differently expressed genes from microarray results selected for validation by RT-qPCR.

Table S7. Sequence of miRNAs primers selected for validation.

Table S8. Sequence of genes primers selected for validation.

Table S1. miRNA sequencing results

	miRNA	logFC	PValue	FDR
**BMFC vs Control**	**miR-146a-3p**	-3.674	0.000	0.013
	**miR-3552**	-3.389	0.000	0.013
	**miR-181a-5p**	-2.760	0.001	0.039
	**miR-17-1-3p**	-2.168	0.001	0.032
	**miR-483-3p**	-2.083	0.001	0.032
	**miR-17-5p**	-1.756	0.001	0.033
	**miR-20a-5p**	-1.575	0.000	0.013
	**miR-339-5p**	-1.569	0.000	0.013
	**miR-99a-3p**	-1.501	0.001	0.032
	**miR-3102**	-1.397	0.000	0.008
	**miR-31a-5p**	-1.394	0.001	0.033
	**miR-582-5p**	-1.376	0.001	0.032
	**miR-3065-3p**	-1.261	0.001	0.035
	**miR-369-3p**	-1.255	0.002	0.044
	**miR-872-3p**	-1.238	0.001	0.039
	**miR-219a-5p**	-1.147	0.001	0.032
	**miR-674-5p**	-1.004	0.002	0.041
	**miR-450a-5p**	-0.970	0.001	0.032
	**miR-15b-5p**	-0.956	0.002	0.044
	**miR-181b-5p**	-0.932	0.001	0.040
	**let-7f-5p**	0.822	0.002	0.045
	**miR-148a-5p**	0.918	0.002	0.047
	**miR-195-3p**	1.023	0.002	0.042

	miRNA	logFC	PValue	FDR
	**miR-17-5p**	-2.747	0.001	0.008
	**miR-3583-3p**	-2.575	0.011	0.045
	**miR-362-3p**	-2.464	0.002	0.013
	**miR-17-1-3p**	-2.377	0.007	0.031
	**miR-20a-5p**	-2.305	0.000	0.000
	**miR-449a-5p**	-2.287	0.002	0.014
	**miR-369-3p**	-2.254	0.000	0.006
	**miR-362-5p**	-2.242	0.001	0.009
	**let-7f-2-3p**	-2.192	0.002	0.015
	**miR-339-5p**	-2.154	0.000	0.004
	**miR-764-3p**	-2.115	0.011	0.043
	**miR-107-3p**	-2.102	0.000	0.002
	**miR-194-5p**	-1.922	0.000	0.002
	**miR-770-5p**	-1.899	0.001	0.009
	**miR-144-5p**	-1.896	0.006	0.028
	**miR-93-3p**	-1.876	0.003	0.018
	**miR-374-5p**	-1.849	0.000	0.001
	**miR-582-5p**	-1.824	0.000	0.003
	**miR-3065-3p**	-1.797	0.000	0.002
	**miR-137-3p**	-1.771	0.000	0.002
	**miR-99a-3p**	-1.699	0.001	0.008
	**miR-106b-5p**	-1.696	0.000	0.001
	**miR-324-3p**	-1.689	0.000	0.005
	**miR-539-3p**	-1.671	0.000	0.006
	**miR-143-5p**	-1.592	0.001	0.008
	**miR-34c-3p**	-1.585	0.003	0.017
	**miR-30c-5p**	-1.546	0.001	0.007
	**miR-494-3p**	-1.543	0.000	0.006
	**miR-872-3p**	-1.522	0.001	0.009

BMFC+KOC vs Control	**miR-98-3p**	-1.465	0.009	0.038
	**miR-31a-5p**	-1.457	0.000	0.004
	**miR-16-5p**	-1.453	0.000	0.002
	**miR-126a-5p**	-1.408	0.001	0.008
	**miR-708-5p**	-1.396	0.000	0.005
	**miR-410-3p**	-1.391	0.000	0.006
	**miR-1193-3p**	-1.344	0.008	0.033
	**miR-425-3p**	-1.326	0.002	0.015
	**miR-93-5p**	-1.272	0.000	0.004
	**miR-674-5p**	-1.266	0.001	0.008
	**miR-376b-3p**	-1.238	0.004	0.022
	**miR-361-5p**	-1.225	0.001	0.009
	**miR-181b-5p**	-1.222	0.001	0.009
	**miR-221-3p**	-1.216	0.001	0.008
	**miR-219a-5p**	-1.200	0.007	0.033
	**miR-450a-5p**	-1.181	0.001	0.008
	**miR-186-5p**	-1.177	0.000	0.006
	**miR-138-5p**	-1.166	0.001	0.009
	**miR-497-5p**	-1.146	0.007	0.029
	**miR-3589**	-1.126	0.003	0.019
	**let-7e-3p**	-1.079	0.001	0.009
	**miR-384-5p**	-1.073	0.004	0.022
	**miR-98-5p**	-1.071	0.001	0.009
	**miR-15b-5p**	-1.048	0.006	0.027
	**miR-384-3p**	-0.997	0.006	0.028
	**miR-874-3p**	-0.986	0.004	0.023
	**miR-379-3p**	-0.970	0.002	0.015
	**miR-23a-3p**	-0.892	0.006	0.028
	**miR-195-5p**	-0.891	0.006	0.028
	**miR-191a-5p**	-0.874	0.003	0.016
	**miR-140-5p**	-0.863	0.003	0.019
	**miR-421-3p**	-0.863	0.009	0.038
	**miR-26b-5p**	-0.848	0.004	0.022
	**miR-146a-5p**	-0.844	0.004	0.021
	**miR-29a-3p**	-0.835	0.007	0.033
	**miR-543-3p**	0.690	0.012	0.047
	**miR-455-5p**	0.763	0.012	0.047
	**miR-495**	0.810	0.006	0.028
	**let-7i-5p**	0.811	0.007	0.030
	**miR-541-5p**	0.822	0.009	0.038
	**miR-134-5p**	0.840	0.010	0.041
	**miR-143-3p**	0.884	0.005	0.024
	**miR-135a-3p**	0.899	0.008	0.033
	**miR-125-1-3p**	0.899	0.009	0.038
	**let-7f-5p**	0.905	0.005	0.027
	**miR-139-5p**	0.923	0.001	0.009
	**miR-99a-5p**	0.940	0.004	0.021
	**miR-361-3p**	0.965	0.006	0.028
	**miR-185-3p**	0.969	0.009	0.038
	**miR-99b-5p**	0.973	0.010	0.038
	**miR-380-5p**	0.975	0.005	0.023
	**miR-6331**	0.976	0.004	0.021
	**miR-128-3p**	0.981	0.003	0.018
	**miR-30c-2-3p**	0.995	0.001	0.010
	**miR-1843-5p**	1.008	0.005	0.023
	**miR-106b-3p**	1.015	0.003	0.018
	**miR-192-5p**	1.042	0.001	0.010
	**miR-3559-3p**	1.060	0.009	0.038

**miR-1843-5p**	1.092	0.004	0.022
**miR-99b-3p**	1.156	0.001	0.008
**miR-184**	1.180	0.003	0.019
**miR-30e-3p**	1.208	0.002	0.013
**miR-676**	1.227	0.000	0.003
**miR-344-1-3p**	1.236	0.000	0.001
**miR-673-3p**	1.264	0.000	0.004
**miR-770-3p**	1.302	0.000	0.005
**miR-493-3p**	1.320	0.002	0.015
**miR-370-3p**	1.367	0.001	0.009
**miR-485-5p**	1.383	0.001	0.010
**miR-379-5p**	1.393	0.000	0.002
**miR-3557-5p**	1.422	0.000	0.003
**miR-127-3p**	1.423	0.000	0.001
**miR-129-5p**	1.460	0.002	0.015
**miR-219-1-3p**	1.464	0.002	0.012
**miR-150-3p**	1.605	0.001	0.007
**miR-671**	1.615	0.000	0.004
**miR-381-3p**	1.639	0.000	0.002
**miR-674-3p**	1.665	0.000	0.001
**miR-148a-5p**	1.682	0.000	0.001
**miR-6329**	1.690	0.000	0.001
**miR-10a-5p**	1.697	0.002	0.012
**miR-27a-5p**	1.725	0.000	0.002
**miR-409b**	1.728	0.000	0.001
**miR-10b-5p**	1.748	0.002	0.012
**miR-195-3p**	1.829	0.000	0.001
**miR-708-3p**	1.935	0.000	0.000
**miR-6315**	1.984	0.000	0.003
**miR-151-3p**	2.087	0.000	0.004
**miR-148a-3p**	2.288	0.000	0.000
**miR-124-5p**	3.505	0.002	0.013

BMFC: buttermilk fat concentrate rich in phospho- and sphingolipids; KOC: krill oil concentrate rich in omega-3 fatty acids (eicosapentaenoic acid and docosahexaenoic acid) and phospholipids; BMFC+KOC: combination of BMFC and KOC.

Table S2. Selected miRNAs for validation by RT-qPCR.

	miRNA	logFC	PValue	FDR	Function
**BMFC**	**let-7f-5p**	0.822	0.002	0.045	Down-regulation in AD
	**miR-195-3p**	1.023	0.002	0.042	Role in determining dementia susceptibility
	**miR-148a-5p**	0.918	0.002	0.047	Inhibition induces hepatocellular tumorigenesis
**BMFC+KOC**	**miR-99a-5p**	0.940	0.004	0.021	Neuroprotective effect
	**miR-128-3p**	0.981	0.003	0.018	Critical for hippocampus-related contextual learning
	**miR-148a-3p**	2.288	0.000	0.000	Downregulation in AD
	**miR-29a-3p**	-0.835	0.007	0.033	Neuronal synapse formation and plasticity
	**miR-191a-5p**	-0.874	0.003	0.016	Target genes to apoptosis and cell death pathways
	**miR-381-3p**	1.639	0.000	0.002	Linked to insulin resistance or T2D
	**miR-379-5p**	1.393	0.000	0.002	Predispose to neuropathic pain identified
	**miR-30e-3p**	1.208	0.002	0.013	Linked to insulin resistance
	**miR-146a-5p**	-0.844	0.004	0.021	Associated with AD.
	**miR-370-3p**	1.367	0.001	0.009	Downregulated by Mild Stress in Rat Hippocampal Tissues
	**let-7f-5p**	0.905	0.005	0.027	Downregulation in AD
	**miR-770-3p**	1.302	0.000	0.005	Down-regulated in the hippocampus of Sprague-Dawley rats with temporal lobe epilepsy
	**miR-106b-3p**	1.015	0.003	0.018	Protect mitochondrial functions
	**miR-195-3p**	1.829	0.000	0.001	Downregulation in AD
	**miR-148a-5p**	1.682	0.000	0.001	Cancer

BMFC: buttermilk fat concentrate rich in phospho- and sphingolipids; KOC: krill oil concentrate rich in omega-3 fatty acids (eicosapentaenoic acid and docosahexaenoic acid) and phospholipids; BMFC+KOC: combination of BMFC and KOC.

Table S3 (see Excel file attached to this manuscript supporting information)

Table S4. Gene expression microarray results.

	Gene	logFC	P.Value	Adj.P.Val
**KOC**	**Kcnj13**	-3	0.0000	0.0315
	**Dpt**	-2.4	0.0000	0.0275
	**Tph1**	-2.1	0.0000	0.0000
	**Slc35f4**	-2	0.0001	0.0452
	**Exoc5**	-1.8	0.0000	0.0315
	**Barx1**	-1.7	0.0000	0.0005
	**Ippk**	-1.5	0.0000	0.0013
	**Lilrb3l**	-1.4	0.0000	0.0013
	**P4hb**	-1.3	0.0000	0.0008
	**Cenpn**	-1.3	0.0001	0.0438
	**---**	-1.2	0.0000	0.0049
	**Enpp2**	-1.2	0.0001	0.0380
	**Osr1**	-1.1	0.0000	0.0275
	**Ptgds**	-1.1	0.0000	0.0315
	**LOC654482**	-1.1	0.0001	0.0341
	**Slc6a20**	-1.1	0.0001	0.0380
	**Bicd2**	-1	0.0000	0.0226
	**Ch25h**	-1	0.0000	0.0293
	**Arhgef15**	-1	0.0001	0.0371
	**Sox21**	-1	0.0001	0.0452
	**Nutm2f**	-1	0.0001	0.0452
	**Rarres1**	-0.9	0.0000	0.0315
	**Car13**	-0.9	0.0001	0.0332
	**Id4**	-0.9	0.0001	0.0438
	**Efhc1**	-0.9	0.0002	0.0490
	**Ctsc**	-0.8	0.0001	0.0452
	**Aox4**	-0.8	0.0001	0.0452
	**Gpr135**	0.8	0.0001	0.0426
	**Pygo2**	0.8	0.0001	0.0452
	**Fam163b**	0.8	0.0001	0.0452
	**Ptpn4**	0.8	0.0001	0.0452
	**St6gal2**	0.8	0.0002	0.0495
	**Pak6**	0.9	0.0000	0.0315
	**Arc**	0.9	0.0000	0.0315
	**Ramp3**	0.9	0.0001	0.0341
	**Cckbr**	0.9	0.0001	0.0371
	**N4bp2**	1	0.0000	0.0266
	**Yeats2**	1	0.0001	0.0438
**BMFC**	**Kcnj13**	-2.8	2.53E-12	2.91E-08
	**Dpt**	-2.3	8.23E-11	4.74E-07
	**Zfp358**	-2.1	4.15E-09	6.81E-06
	**Cbr3**	-2	2.60E-09	4.99E-06

**Mfrp**	-2	1.51E-08	2.17E-05
**Tmem72**	-1.9	5.70E-08	7.29E-05
**Sostdc1**	-1.8	4.66E-10	1.34E-06
**Barx1**	-1.8	4.56E-10	1.34E-06
**Ippk**	-1.6	2.29E-09	4.99E-06
**Slco1a5**	-1.6	1.05E-07	0.00011518
**Tph1**	-1.6	2.45E-06	0.0011723
**Ahsg**	-1.5	3.48E-07	0.00026687
**Slc35f4**	-1.4	1.10E-07	0.00011518
**Exoc5**	-1.4	1.75E-07	0.00016792
**Slc4a5**	-1.4	2.40E-07	0.00020184
**Slc13a4**	-1.4	4.28E-07	0.00029527
**Prlr**	-1.4	1.45E-05	0.00476127
**Ict1**	-1.3	2.46E-07	0.00020184
**C7**	-1.3	4.88E-07	0.00029527
**Prr32**	-1.3	6.79E-07	0.00039051
**Zfp24**	-1.3	6.74E-06	0.0025839
**Ptgds**	-1.2	4.47E-07	0.00029527
**---**	-1.2	4.70E-07	0.00029527
**Slc6a20**	-1.2	7.25E-07	0.00039735
**Aqp1**	-1.2	7.68E-06	0.00276169
**Cldn2**	-1.2	5.98E-05	0.01465844
**Myog**	-1.1	1.60E-06	0.00079918
**Ctsc**	-1.1	3.03E-06	0.00134359
**Enpp2**	-1.1	3.04E-06	0.00134359
**Ch25h**	-1.1	3.43E-06	0.00146244
**Abca4**	-1.1	4.89E-06	0.00200889
**Folr1**	-1.1	2.45E-05	0.00721838
**Cbr1**	-1.1	5.20E-05	0.01329863
**Emp3**	-1	6.23E-06	0.00247225
**Bicd2**	-1	1.24E-05	0.00430572
**LOC654482**	-1	1.31E-05	0.0044311
**Mospd1**	-1	1.67E-05	0.00533843
**Lepr**	-1	1.77E-05	0.00551727
**P4hb**	-1	2.09E-05	0.00632722
**Atp11c**	-1	3.81E-05	0.01086025
**Cenpn**	-1	4.38E-05	0.01184456
**Igf2**	-1	6.44E-05	0.01544012
**Rpl9**	-1	8.19E-05	0.01884002
**Sst**	-1	8.66E-05	0.01952348
**Tmem27**	-1	0.000134	0.02854155
**Elovl7**	-0.9	3.87E-05	0.01086025
**Pla2g5**	-0.9	4.43E-05	0.01184456
**Ranbp3l**	-0.9	4.77E-05	0.01247554
**Hmgb2l1**	-0.9	5.99E-05	0.01465844

	**Trim34**	-0.9	6.94E-05	0.01628543
	**Capn7**	-0.9	0.00011415	0.02525007
	**Slc2a12**	-0.9	0.00013173	0.02854155
	**C1qtnf3**	-0.9	0.00014208	0.02971226
	**Mdk**	-0.9	0.00014941	0.03068729
	**Slc22a8**	-0.8	0.00021362	0.04310616
	**Spata20**	-0.8	0.00022686	0.04498921
	**Arc**	1	7.18E-06	0.00266579
	**Adss**	1.3	1.03E-06	0.00053942
**BMFC+KOC**	**Zfp358**	-3.6	0.0000	0.0000
	**Kcnj13**	-3.9	0.0000	0.0000
	**Dpt**	-3.7	0.0000	0.0000
	**Zfp24**	-3.5	0.0000	0.0000
	**Cbr3**	-3.4	0.0000	0.0000
	**Mfrp**	-3.2	0.0000	0.0000
	**Sostdc1**	-2.9	0.0000	0.0000
	**Tmem72**	-2.9	0.0000	0.0000
	**Prlr**	-2.6	0.0000	0.0000
	**Ahsg**	-2.4	0.0000	0.0000
	**Exoc5**	-2.4	0.0000	0.0000
	**Cldn2**	-2.4	0.0000	0.0001
	**Slc35f4**	-2.2	0.0000	0.0000
	**Slc4a5**	-2.1	0.0000	0.0000
	**Folr1**	-2	0.0000	0.0000
	**Prr32**	-2	0.0000	0.0000
	**Slco1a5**	-2	0.0000	0.0000
	**Ict1**	-1.9	0.0000	0.0000
	**Tmem27**	-1.8	0.0000	0.0010
	**Sst**	-1.6	0.0000	0.0011
	**Cenpn**	-1.5	0.0000	0.0002
	**Aqp1**	-1.5	0.0000	0.0007
	**Abca4**	-1.5	0.0000	0.0007
	**Cbr1**	-1.5	0.0000	0.0022
	**Slc13a4**	-1.5	0.0000	0.0117
	**Htr2c**	-1.4	0.0000	0.0008
	**Capn7**	-1.3	0.0000	0.0014
	**Itpripl1**	-1.2	0.0000	0.0015
	**Glycam1**	-1.2	0.0000	0.0015
	**Pla2g5**	-1.2	0.0000	0.0017
	**LOC654482**	-1.2	0.0000	0.0022
	**Rpl9**	-1.2	0.0000	0.0063
	**Enpp2**	-1.1	0.0000	0.0021
	**Spata20**	-1.1	0.0000	0.0044
	**Itgb6**	-1.1	0.0000	0.0057
	**Atp11c**	-1.1	0.0000	0.0070

**Scgb1c1**	-1.1	0.0000	0.0077
**Aimp2**	-1.1	0.0000	0.0098
**Mdk**	-1.1	0.0001	0.0231
**Slc2a12**	-1.1	0.0003	0.0475
**Efhc1**	-1	0.0000	0.0075
**Tbc1d10a**	-1	0.0000	0.0088
**Creg1**	-1	0.0001	0.0177
**Diaph3**	-1	0.0001	0.0239
**Narfl**	-0.9	0.0000	0.0082
**Wdr63**	-0.9	0.0000	0.0100
**RGD1564074**	-0.9	0.0000	0.0111
**Glipr2**	-0.9	0.0000	0.0117
**Fbxo45**	-0.9	0.0000	0.0119
**LOC100909960**	-0.9	0.0001	0.0134
**Lama2**	-0.9	0.0001	0.0163
**Cars2**	-0.9	0.0001	0.0264
**Olr109**	-0.9	0.0001	0.0289
**Slc4a2**	-0.9	0.0002	0.0316
**Wfs1**	-0.9	0.0002	0.0365
**Muc19l1**	-0.8	0.0001	0.0239
**Igfbpl1**	-0.8	0.0002	0.0383
**Mast3**	-0.8	0.0002	0.0384
**---**	-0.8	0.0002	0.0415
**Fam92b**	-0.8	0.0003	0.0473
**Six3**	-0.8	0.0003	0.0486
**LOC100911887**	0.8	0.0001	0.0276
**Samd15**	0.8	0.0002	0.0365
**Tnks**	0.8	0.0003	0.0465
**Sptssb**	0.8	0.0003	0.0469
**Satb2**	0.8	0.0003	0.0489
**ATP6**	0.8	0.0003	0.0489
**MGC116197**	0.9	0.0002	0.0342
**LOC102550585**	1	0.0000	0.0068
**Cckbr**	1	0.0000	0.0070
**LOC102555217**	1	0.0000	0.0082
**Npas4**	1	0.0002	0.0351

BMFC: buttermilk fat concentrate rich in phospho- and sphingolipids; KOC: krill oil concentrate rich in omega-3 fatty acids (eicosapentaenoic acid and docosahexaenoic acid) and phospholipids; BMFC+KOC: combination of BMFC and KOC.

Table S5 (see Excel file attached to this manuscript supporting information)

Table S6. Differently expressed genes from microarray results selected for validation by RT-qPCR.

Gene	miR Interaction	Study group	Gene Function	miRNA Function
**Arc**	7f-5p	KOC	Neuronal plasticity and memory	Downregulation in AD
**Cbr3**	191a-5p	BMFC	Parkinson's disease	Target genes to apoptosis and cell death pathways
	106b	BMFC+KOC	Parkinson's disease.	Protect mitochondrial functions
**tshz3**	148a-5p	BMFC+KOC	Reduced expression of this gene and consequent caspase upregulation may be correlated with progression of Alzheimer's disease	Inhibition induces hepatocellular tumorigenesis
	195-3p			Downregulation in AD
	30e-3p			Linked to insulin resistance
	381-3p			
**pla2g5**	106b	BMFC+KOC	Increased in several neurological and neurodegenerative disorders	Protect mitochondrial functions
	148a-3p			Downregulation in AD
	195-3p			Downregulation in AD
**htr2c**	195-3p	BMFC+KOC	Signaling of neurotransmitters	Downregulation in AD
	379-5p			Predispose to neuropathic pain identified
**Mrps23**	379-5p	KOC	Mitochondrial ribosomal proteins	Predispose to neuropathic pain identified
**Capn7**	128-3p	BMFC	Calpains are ubiquitous, have been implicated in neurodegenerative processes,	Critical for hippocampus-related contextual learning
	370-3p	KOC		Downregulated by Mild Stress in Rat Hippocampal Tissues
	128-3p	BMFC+KOC		Critical for hippocampus-related contextual learning
	370-3p			Downregulated by Mild Stress in Rat Hippocampal Tissues

	381-3p			Linked to insulin resistance or T2D
**Sst**	106b	BMFC+KOC	Neurotransmission	Protect mitochondrial functions
**Satb2**	128-3p	BMFC+KOC	Memory	Critical for hippocampus-related contextual learning
	148a-5p			Inhibition induces hepatocellular tumorigenesis
	381-3p			Linked to insulin resistance or T2D
**Ip6k1**	148a-5p	BMFC+KOC	Neurotransmission	Inhibition induces hepatocellular tumorigenesis
	770-3p			Down-regulated in the hippocampus of Sprague-Dawley rats with temporal lobe epilepsy
**Tph1**	379-5p	BMFC & KOC	biosynthesis of serotonin	Identified in primary sensory neurons that are associated with neuropathic pain

Arc: activity-regulated cytoskeleton-associated protein; Cbr3: carbonyl reductase 3; Tshz3: teashirt zinc finger homeobox 3; Pla2g5: phospholipase A2, group V; Htr2c: 5-hydroxytryptamine receptor 2C; Mrps23: mitochondrial ribosomal protein S23; Capn7: calpain 7; Sst: somatostatin; Satb2: SATB homeobox 2; Ip6k1: inositol hexakisphosphate kinase 1; Tph1: tryptophan hydroxylase 1; BMFC: buttermilk fat concentrate rich in phospho- and sphingolipids; KOC: krill oil concentrate rich in omega-3 fatty acids (eicosapentaenoic acid and docosahexaenoic acid) and phospholipids; BMFC+KOC: combination of BMFC and KOC.

Table S7. Sequence of miRNAs primers selected for validation.

**miRNA**	**sequence**
rno-let-7f-5p	TGAGGTAGTAGATTGTATAGTT
rno-miR-106b-3p	CCGCACTGTGGGTACTTGCTGC
rno-miR-128-3p	TCACAGTGAACCGGTCTCTTT
rno-miR-146a-5p	TGAGAACTGAATTCCATGGGTT
rno-miR-148a-3p	TCAGTGCACTACAGAACTTTG
rno-miR-148a-5p	AAAGTTCTGAGACACTCTGACTC
rno-miR-191a-5p	CAACGGAATCCCAAAAGCAGCTG
rno-miR-195-3p	CCAATATTGGCTGTGCTGCTCCA
rno-miR-29a-3p	TAGCACCATCTGAAATCGGTTA
rno-miR-30e-3p	CTTTCAGTCGGATGTTTACAGC
rno-miR-370-3p	GCCTGCTGGGGTGGAACCTGGT
rno-miR-381-3p	TATACAAGGGCAAGCTCT
rno-miR-770-3p	GTGGGCCTGACGTGGAG
rno-miR-99a-5p	AACCCGTAGATCCGATCTTGTG
rno-miR-379-5p	TGGTAGACTATGGAACGTAGG

Table S8. Sequence of genes primers selected for validation.

Gene	Name	Sequence
**Arc**	rno-Arc-FW	AAAGCAGCAGCAAGATGGTT
	rno-Arc-REV	GAGTCTTGCCTCCTGTCCTG
**Cbr3**	rno-Cbr3-FW	GCTGCCCATAATGAAACCACA
	rno-Cbr3-REV	GTCTGGCCAACCTTCTCTCT
**Tshz3**	rno-Tshz3-FW	GCAGCACAGCCATTATCACG
	rno-Tshz3-REV	GGCCAGACTGTTGCTCATCT
**Pla2g5**	rno-Pla2g5-FW	CTTGGGCTGCCAGCATAAAC
	rno-Pla2g5-REV	GCAGCCGTAGAAGCCATAGT
**Htr2c**	rno-Htr2c-FW	CGGACGGGGTACAAAACTGG
	rno-Htr2c-REV	AATCCAGACGGGGCACAAAT
**Mrps23**	rno-Mrps23-FW	CACATGGTGTGGTTCCTCGG
	rno-Mrps23-REV	CTTAGCCCAACCCGTGACAT
**Capn7**	rno-Capn7-FW	AGTTCTCCTCGCAGTGCCTC
	rno-Capn7-REV	TTGGGCAGCTTCCTTGTAGTA
**Sst**	rno-Sst-FW	ACCCCAGACTCCGTCAGTTT
	rno-Sst -REV	CCAGGGCATCGTTCTCTGTC
**Satb2**	rno-Satb2-FW	AAAACCTCGACACCGACAAC
	rno-Satb2-REV	CCAACGAAGCAGTTCACAGA
**Ip6k1**	rno-Ip6k1-FW	ACCAAGGCTGCATCATTTTGAC
	rno-Ip6k1-REV	AAACACACATTGCGTTGGGG

Arc: activity-regulated cytoskeleton-associated protein; Cbr3: carbonyl reductase 3; Tshz3: teashirt zinc finger homeobox 3; Pla2g5: phospholipase A2, group V; Htr2c: 5-hydroxytryptamine receptor 2C; Mrps23: mitochondrial ribosomal protein S23; Capn7: calpain 7; Sst: somatostatin; Satb2: SATB homeobox 2; Ip6k1: inositol hexakisphosphate kinase 1; Tph1: tryptophan hydroxylase 1; FW: Forward; REV: Reverse.
